# Adolescent Carers' Psychological Symptoms and Mental Well-being During the COVID-19 Pandemic: Longitudinal Study Using Data From the UK Millennium Cohort Study

**DOI:** 10.1016/j.jadohealth.2022.01.228

**Published:** 2022-06

**Authors:** Miharu Nakanishi, Marcus Richards, Daniel Stanyon, Syudo Yamasaki, Kaori Endo, Mai Sakai, Hatsumi Yoshii, Atsushi Nishida

**Affiliations:** a Department of Psychiatric Nursing, Tohoku University Graduate School of Medicine, Sendai-shi, Miyagi, Japan; b Research Center for Social Science & Medicine, Tokyo Metropolitan Institute of Medical Science, Setagaya-ku, Tokyo, Japan; c MRC Unit for Lifelong Health and Ageing at UCL, University College London, London, United Kingdom

**Keywords:** Adolescent, COVID-19, Family caregiving, Mental well-being, Psychological, Symptoms, Young carers

## Abstract

**Purpose:**

During the COVID-19 pandemic, adolescent carers in the UK may have experienced psychological distress owing to increased caring burden and loss of a break from their caring role. This study investigated longitudinal association between adolescents' caring status and mental health outcomes from 2018/2019 to February–March 2021.

**Methods:**

The participants (n = 3,927) answered mental health questions in both the Millennium Cohort Study sweep 7 survey (age 17 years in 2018/2019) and at least one of three waves of the COVID-19 survey from May 2020 to February–March 2021. Caring status at the age of 17 years was assessed using a single question regarding whether the participant regularly looked after anyone who needed care, without being paid. Outcome measures were psychological symptoms, measured using the Kessler Distress Scale, and mental well-being, measured using the Warwick-Edinburgh Mental Well-being Scale.

**Results:**

Compared with 3,616 noncarers, 311 (7.9%) adolescent carers reported significantly higher Kessler Distress Scale and lower Warwick-Edinburgh Mental Well-being Scale scores during the pandemic. These associations were largely explained by psychosocial risk factors. Worse outcomes were associated with poor sleep quality, attempted suicide at baseline, low social support, and a strong feeling of loneliness during the pandemic. These factors were significantly more likely to be observed among adolescent carers than noncarers.

**Discussion:**

UK adolescent carers exhibited worsened mental health outcomes one year after the first national lockdown. This increased distress may be attributable to psychosocial risk factors during the pre–COVID-19 and current COVID-19 periods, and they require psychosocial support.

**Implications and Contribution**

There are long-term impacts of the pandemic on adolescent carers' mental health outcomes. This highlights the need for psychosocial support for young carers. Careful consideration is warranted to support adolescent carers to return to school and catch up with studies and other students.

The impact of the COVID-19 pandemic and related restrictions is concerning for the mental health of adolescents [1]. The restrictions include self-isolation, physical distancing, and closure of places involving social gatherings. These restrictions have resulted in significant disruptions to daily life, the education system, and health service delivery [2]. There has been evidence of increases in the prevalence of mental health problems among young people during the pandemic [3]. Adolescence is a formative period for emotional and social development [4,5], which is facilitated by social connections and peer interactions [6]. Thus, school closures and stay-at-home orders have been linked to adolescent mental health problems [7]. The first lockdown across the UK was announced on 23 March 2020; subsequently, COVID-19 cases resurged after ease of restrictions which led to two further national lockdowns over one year. These long-term public health restrictions can cause chronic psychological distress and negative effects on social and emotional development in adolescence. However, most reports focus on a few months after the first national lockdown [8]. Therefore, little is known about the long-term consequences of COVID-19 on adolescent mental health.

In particular, adolescent carers in the UK have been facing growing pressure during the pandemic. They undertake a range of tasks to support family members or friends who cannot cope without their support. Caring responsibilities in adolescence may have serious impact on psychosocial adjustment and represent a challenge to life planning in relation to education, career, and personal life [9]. There are an estimated number of 800,000 young carers in the UK under the age of 18 years [10]. Mental health needs of young carers have been recognized prior to the COVID-19 pandemic. In the UK, young carers were more likely to report psychosocial difficulties and mental health problems [11]. Of them, 45% reported having some form of mental health problem [12]. During the COVID-19 pandemic, most adolescent carers in the UK have experienced psychological distress owing to lack of school and established routines, increased caring burden, and loss of a break from home and their caring role [13,14]. However, there has been a lack of awareness and visibility concerning these adolescent carers in most European countries [[15], [16], [17]]. Furthermore, there has been no longitudinal examination of the mental health of adolescent carers from the pre–COVID-19 to current COVID-19 period in comparison with the general population. Such an understanding will highlight social inequalities in the context of the COVID-19 pandemic and help identify urgent support needs in the adolescent population.

This study aimed to investigate the longitudinal association between adolescents' caring status and mental health outcomes among adolescents from pre–COVID-19 to current COVID-19 periods. Since we expected that adolescent carers were more likely to experience psychosocial risk factors before and during COVID-19 periods, we conducted hierarchical regression analyses. To determine caring role and psychosocial factors before and during COVID-19 periods associated with mental health outcomes, the unadjusted model included caring status. The second model included pre–COVID-19 psychosocial factors, and the fully adjusted model included psychosocial factors at current COVID-19 periods.

## Methods

### Study design and participants

The sample for this study was drawn from the Millennium Cohort Study (MCS) [18]. The MCS is a nationally representative birth cohort study following the lives of 18,818 people born across England, Scotland, Wales, and Northern Ireland in 2000–2002. The MCS collects developmental information on physical and mental health throughout childhood and adolescence. The most recent sweep (sweep 7) was conducted when the cohort members were 17 years old, from January 2018 to March 2019.

In May 2020, the COVID-19 survey was launched in five national longitudinal studies, including the MCS. The survey aimed to explore the effect of the pandemic on the lives of people of different generations and backgrounds [19,20].

We combined data from the MCS sweep 7 survey (baseline) and the COVID-19 survey (exposure) administered to MCS members. Data available from the COVID-19 survey included waves 1–3. The wave 1 survey was conducted at the height of lockdown restrictions in May 2020. The wave 2 survey was conducted in September and October 2020 and focused on the period of restriction easing. The wave 3 survey was conducted in February and March of 2021. During this period, a road map for easing lockdown restrictions was announced in the UK. A summary of the data sources is provided in the Appendix A1.

The participants varied based on the waves they were sampled in and the number of times they participated. A total of 3,927 participants completed the mental health questions in the MCS sweep 7 and in least one wave of the COVID-19 survey. This sample was used in the present study.

### Data collection and procedures

Ethical approval was not required for this study. All secondary data used in this study were drawn from the MCS and COVID-19 survey and were anonymized at the sources by the survey team. Consequently, no data were collected directly from human subjects in the present study. However, all the participants provided written informed consent for the original data collection.

### Measures

Our primary outcome measures were psychological symptoms and mental well-being. Psychological symptoms were measured using the Kessler Distress Scale (K6) [21]. It contains six items to rate each symptom in the last 30 days on a 5-point Likert scale. Mental well-being was measured using the Warwick-Edinburgh Mental Well-being Scale (WEMWBS) [22]. It contains seven items to rate each condition over the past two weeks on a 5-point Likert scale. The K6 and WEMWBS were evaluated at baseline and at each wave of the COVID-19 survey.

The primary explanatory measure was caring status at baseline. The MCS sweep 7 survey online questionnaire asked the following question: ‘Do you regularly look after anyone who is ill, disabled, or elderly and in need of care, without being paid? This includes both people who live with you and those who live elsewhere. Please do not include caring for others that you do in a professional capacity (i.e., as a job)’. Participants who responded ‘yes’ and ‘no’ were categorized as carers and noncarers, respectively.

The covariates in this study were demographic variables at baseline and psychosocial risk factors at baseline and after exposure to the COVID-19 pandemic. These covariates were selected based on a previous study using the MCS sweep 7 survey and COVID-19 wave 1 survey [23]. Details of the variables are available in the Appendix A1. Demographic variables included age, sex, and ethnicity. The psychosocial variables at baseline were heavy drinking, regular smoking, cannabis use, use of other drugs, arrested by a police officer, problematic video gaming, gambling, poor sleep quality, self-harm, suicide attempts, and mental difficulties. Mental difficulties were evaluated by parents using the Strength and Difficulties Questionnaire [24]. The Strength and Difficulties Questionnaire contains 25 items to measure adolescents' strengths and difficulties on a 3-point Likert scale. Psychosocial variables after exposure to the COVID-19 pandemic included sleep time, smoking (number of cigarettes per day), alcohol consumption, outdoor spaces at home, financial management, changes in household composition, social support, and feelings of loneliness. Social support was measured using a 3-item version of the Social Provisions Scale [25]. This assesses the availability of social support on a 3-point Likert scale. Loneliness was measured using a 4-item UCLA Loneliness Scale [26]; items are rated on a 3-point Likert scale.

### Data analyses

Baseline demographic and psychosocial variables were compared between carers and noncarers. Differences in psychosocial variables after exposure were also examined as per the caring status. Student's t-tests were used for continuous variables, Mann-Whitney's U tests were used for ranked variables, and χ^2^ tests were used for categorical variables.

To determine the association between caring status and outcome measures, a multivariable linear regression analysis was performed. The independent variables comprised caring status at baseline and time (wave 1 vs. wave 2 or 3). The corresponding outcome measure at baseline (i.e., psychological symptoms or mental well-being) was included in the model as a covariate. Three models were generated: unadjusted, adjusted for demographic and psychosocial variables at baseline, and adjusted for baseline features and psychosocial variables after exposure to the COVID-19 pandemic. In these analyses, each case had a time variable (wave 1, wave 2, or wave 3) and variables at baseline and the time of assessment. These models accounted for the clustering of outcome measures among adolescents. As our concerns were not about the clustering level but controlling for within-cluster correlation, we used a sandwich estimator instead of modeling random effects [27]. To assess how much the covariable adjustments change the effect sizes of associations, the local effect size per model was calculated using Cohen's f^2^. The effect size was considered small if f^2^ values varied by approximately .02, medium if approximately .15, and large if more than .35 [28].

In the regression analysis, full information maximum likelihood was used to estimate the missing data [29]. To ensure robustness of the missing data, a sensitivity analysis of the fully adjusted model was performed by excluding individuals with missing data. All analyses were conducted using Mplus for Windows, version 8.4 (Muthén & Muthén, Los Angeles, California, USA). The statistical significance was set at α = .05.

## Results

### Characteristics of adolescent carers

At baseline, there were 311 carers (7.9%) among the 3,927 adolescents. Compared with noncarers, adolescent carers were significantly younger; included more non-White ethnicities; had poorer sleep quality and more severe mental difficulties; and had a higher frequency of gambling, smoking, self-harm, and suicide attempts (Table 1).

**Table 1:** Adolescent characteristics at baseline by caring status

MCS sweep 7 survey, at the age of 17 years	Carer	N (%) or mean (SD)	Noncarer	N (%) or mean (SD)	Test statistic	*p* value
Demographic						
Age, year, mean (SD)	N = 311	17.1 (0.3)	N = 3,615	17.2 (0.3)	t(373.11) = 2.16∗	.031
Female, N (%)	N = 310	200 (64.5)	N = 3,594	2,218 (61.7)	χ^2^(1) = 0.95	.330
Non-White/non-Caucasian, N (%)	N = 289	66 (22.8)	N = 3,457	548 (15.9)	χ^2^(1) = 9.50∗	.002
Psychosocial risk factors						
Heavy drinking, N (%)	N = 210	23 (11.0)	N = 2,511	318 (12.7)	χ^2^(1) = 0.52	.472
Regular smoking, N (%)	N = 311	27 (8.7)	N = 3,611	136 (3.8)	χ^2^(1) = 17.37∗	<.001
Cannabis use, N (%)	N = 311		N = 3,608		Z = 1.15	.250
Never		223 (71.7)		2,691 (74.6)		
Less than 10 times in the last year		70 (22.5)		743 (20.6)		
10 or more times in the last year		18 (5.8)		174 (4.8)		
Use of other drugs, N (%)	N = 308	24 (7.8)	N = 3,580	209 (5.8)	χ^2^(1) = 1.92	.166
Subjective sleep quality, poor, N (%)	N = 311	139 (44.7)	N = 3,615	1,125 (31.1)	χ^2^(1) = 24.17∗	<.001
Being arrested, N (%)	N = 311	6 (1.9)	N = 3,616	32 (0.9)	χ^2^(1) = 3.26	.071
Problematic video gaming, N (%)	N = 311	56 (18.0)	N = 3,616	505 (14.0)	χ^2^(1) = 3.82	.051
Gambling, N (%)	N = 311	49 (15.8)	N = 3,616	407 (11.3)	χ^2^(1) = 5.65∗	.017
Self-harm, N (%)	N = 310	122 (39.4)	N = 3,613	903 (25.0)	χ^2^(1) = 30.51∗	<.001
Suicide attempt, N (%)	N = 311	59 (19.0)	N = 3,608	224 (6.2)	χ^2^(1) = 69.61∗	<.001
Mental difficulties, mean (SD), range 0–40^a^	N = 271	9.4 (6.5)	N = 3,343	6.3 (5.1)	t (297.36) = 7.61∗	<.001

SD = standard deviation.

∗Significant at *p* <·05.

a Mental difficulties were measured using the Strength and Difficulties Questionnaire.

Compared with noncarers, adolescent carers had significantly less sleep time at waves 1 and 3, less frequent outdoor spaces at home at wave 1, lower scores for social support and higher scores for loneliness at all three waves, less alcohol consumption at wave 2 and wave 3, and higher number of cigarettes smoked and worse financial management at wave 3 (Table 2).

**Table 2:** Adolescent characteristics after exposure by caring status

COVID-19 survey	Carer	N (%) or mean (SD)	Noncarer	N (%) or mean (SD)	Test statistic	*p* value
May 2020, wave 1						
Alcohol consumption, number of drinks per day, N (%)	N = 119		N = 1,817		Z = 0.27	.785
Zero		38 (31.9)		543 (29.9)		
1–2		53 (44.5)		838 (46.1)		
3–4		16 (13.4)		287 (15.8)		
5 or more		12 (10.1)		149 (8.2)		
Smoking, number of cigarettes, mean (SD)	N = 118	1.4 (4.4)	N = 1,819	0.6 (2.9)	t (123.63) = 1.77	.079
Sleep time, number of hours, mean (SD)	N = 119	7.6 (2.2)	N = 1,821	8.3 (1.8)	t (127.93) = 3.10∗	.002
Change in household members, N (%)	N = 119	33 (27.7)	N = 1,825	495 (27.1)	χ^2^(1) = 0.02	.885
Outdoor spaces at home, no, N (%)	N = 119	11 (9.2)	N = 1,825	71 (3.9)	χ^2^(1) = 7.92∗	.005
Financial management, worse, N (%)	N = 119	37 (31.0)	N = 1,817	472 (26.0)	χ^2^(1) = 1.51	.219
Social support,^a^ mean (SD), range 3–9	N = 119	8.1 (1.3)	N = 1,820	8.4 (1.0)	t (128.25) = 2.56∗	.012
Loneliness,^b^ mean (SD), range 4–12	N = 119	7.9 (2.3)	N = 1,824	7.1 (2.3)	t (134.05) = 3.71∗	<.001
September/October 2020, wave 2						
Alcohol consumption, number of drinks per day, N (%)	N = 171		N = 2,251		Z = 2.69∗	.007
Never		54 (31.6)		532 (23.6)		
1–2		51 (29.8)		645 (28.7)		
3–4		36 (21.1)		538 (23.9)		
5 or more		30 (17.5)		536 (23.8)		
Smoking, number of cigarettes, mean (SD)	N = 172	2.0 (7.3)	N = 2,225	1.0 (4.3)	t (180.14) = 1.88	.061
Sleep time, number of hours, mean (SD)	N = 171	7.4 (2.0)	N = 2,228	7.6 (1.5)	t (185.57) = 1.24	.218
Change in household members, N (%)	N = 172	65 (37.8)	N = 2,221	725 (32.6)	χ^2^(1) = 1.91	.167
Outdoor spaces at home, no, N (%)	N = 172	26 (15.1)	N = 2,222	340 (15.3)	χ^2^(1) = 0.004	.948
Financial management, worse, N (%)	N = 173	54 (31.2)	N = 2,251	605 (26.9)	χ^2^(1) = 1.53	.217
Social support,^a^ mean (SD), range 3–9	N = 172	8.1 (1.2)	N = 2,251	8.3 (1.1)	t (195.19) = 2.61∗	.010
Loneliness,^b^ mean (SD), range 4–12	N = 172	7.8 (2.4)	N = 2,249	7.0 (2.3)	t (196.32) = 3.97∗	<.001
February/March 2021, wave 3						
Alcohol consumption, number of drinks per day, N (%)	N = 141		N = 1,998		Z = 1.98∗	.048
Never		56 (39.7)		603 (30.2)		
1–2		38 (27.0)		640 (32.0)		
3–4		27 (19.1)		402 (20.1)		
5 or more		20 (14.2)		353 (17.7)		
Smoking, number of cigarettes, mean (SD)	N = 139	2.1 (6.9)	N = 1,985	0.9 (4.7)	t (147.22) = 2.01∗	.047
Sleep time, number of hours, mean (SD)	N = 139	6.9 (1.7)	N = 1,990	7.5 (1.5)	t (153.98) = 4.13∗	<.001
Change in household members, N (%)	N = 257	87 (33.9)	N = 2,945	1,025 (34.8)	χ^2^(1) = 0.09	.758
Outdoor spaces at home, no, N (%)	N = 258	35 (13.6)	N = 2,933	464 (15.8)	χ^2^(1) = 0.91	.339
Financial management, worse, N (%)	N = 257	94 (36.6)	N = 2,988	837 (28.0)	χ^2^(1) = 8.48∗	.004
Social support,^a^ mean (SD), range 3–9	N = 140	7.9 (1.4)	N = 2,008	8.3 (1.2)	t (152.19) = 3.20∗	.002
Loneliness,^b^ mean (SD), range 4–12	N = 256	7.9 (2.5)	N = 2,984	7.2 (2.4)	t (296.36) = 4.46∗	<.001

SD = standard deviation.

∗Significant at *p* <·05.

a Social support was measured using a 3-item version of the Social Provisions Scale.

b Loneliness was measured using a 4-item UCLA Loneliness Scale.

### Psychological symptoms and mental well-being

**Figure 1:**
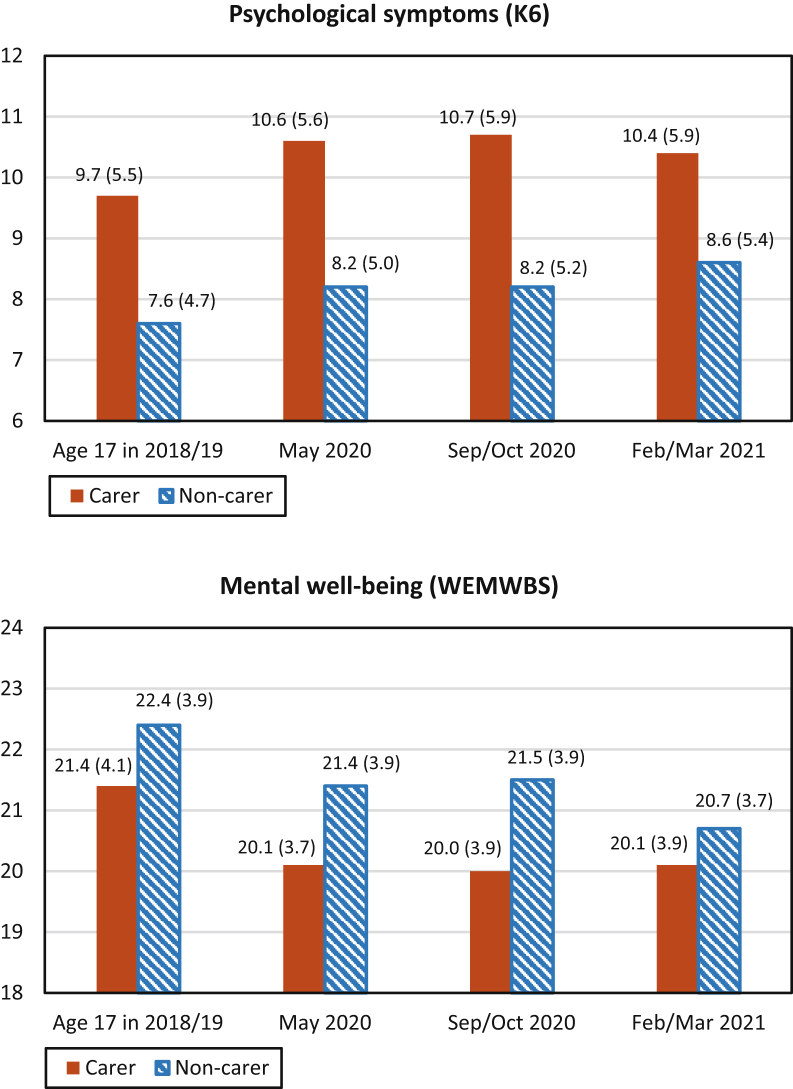
Outcome measures by caring status at each time of assessment. Adolescents aged 17 years in 2018/19 were divided into carers and noncarers. The wave 1 survey was conducted in May 2020, wave 2 was conducted in September–October 2020, and wave 3 was conducted in February–March 2021. The number of participants varied across the assessments. Psychological symptoms were measured using the Kessler Distress Scale (K6; range: 0–24). Mental well-being was measured using the Warwick-Edinburgh Mental Well-being Scale (WEMWBS; range: 7–35).

The mean scores for outcome measures based on caring status at baseline are shown in Figure 1. Carers and noncarers had mean K6 scores of approximately 10 and 8 across the baseline and exposure, respectively. Adolescent carers and noncarers had mean WEMWBS scores of 20–21 during the study period (Figure 1).

### Association between caring status and psychological symptoms

In the unadjusted model, carers showed significantly higher K6 scores than noncarers after exposure (Table 3). The K6 scores at wave 3 were significantly higher than those at wave 1. In the model adjusted for baseline features, caring status remained significantly associated with K6 scores after exposure. The fully adjusted model with psychosocial variables after exposure showed significant associations of greater K6 scores with poor sleep quality, no police arrest, presence of self-harm, suicide attempt at baseline, increased alcohol consumption and smoking, smoking, decreased sleep time and social support, poor financial management, and greater feelings of loneliness, among females. Caring status at baseline was not significantly associated with the psychological symptoms after exposure. The local effect size was small in the unadjusted model and the model adjusted for baseline features. However, it was large in the fully adjusted model (Table 3).

**Table 3:** Multiple linear regression analyses of psychological symptoms controlling for baseline and exposure variables

Coefficient (95% CI)	Unadjusted	Adjusted	Fully adjusted
Carer at baseline	0.86 (0.30, 1.42)∗	0.60 (0.07, 1.13)∗	0.31 (−0.07, 0.69)
Time, reference = wave 1			
Wave 2	0.01 (−0.21, 0.23)	0.07 (−0.14, 0.29)	−0.16 (−0.36, 0.03)
Wave 3	0.47 (0.26, 0.69)∗	0.58 (0.36, 0.79)∗	0.12 (−0.08, 0.31)
Outcome at baseline	0.59 (0.56, 0.62)∗	0.46 (0.42, 0.50)∗	0.27 (0.24, 0.30)∗
Demographic at baseline			
Age, year		−0.22 (−0.62, 0.17)	−0.15 (−0.47, 0.17)
Female		1.35 (1.06, 1.64)∗	1.54 (1.30, 1.77)∗
Non-White Caucasian		−0.09 (−0.48, 0.29)	0.02 (−0.30, 0.33)
Psychosocial risk factors at baseline			
Heavy drinking		0.01 (−0.47, 0.49)	0.14 (−0.29, 0.57)
Regular smoking		0.56 (−0.17, 1.30)	0.23 (−0.39, 0.85)
Cannabis use, reference = never			
Less than 10 times		0.08 (−0.24, 0.41)	0.17 (−0.12, 0.46)
10 or more times		−0.15 (−0.81, 0.51)	0.11 (−0.46, 0.67)
Use of other drugs		−0.12 (−0.75, 0.50)	−0.36 (−0.85, 0.12)
Poor sleep quality		0.91 (0.58, 1.23)∗	0.28 (0.03, 0.53)∗
Being arrested		−0.80 (−2.29, 0.69)	−1.41 (−2.63, −0.19)∗
Problematic video gaming		0.01 (−0.39, 0.41)	−0.09 (−0.44, 0.27)
Gambling		−0.30 (−0.68, 0.09)	−0.22 (−0.52, 0.08)
Self-harm		0.90 (0.56, 1.24)∗	0.37 (0.10, 0.63)∗
Suicide attempt		0.81 (0.22, 1.39)∗	0.89 (0.41, 1.37)∗
Mental difficulties,^a^ range 0–40		0.05 (0.02, 0.07)∗	0.01 (−0.01, 0.04)
Psychosocial risk factors after exposure			
Alcohol consumption, number of drinks per day, reference = never			
1–2			−0.04 (−0.31, 0.23)
3–4			0.35 (0.04, 0.65)∗
5 or more			0.64 (0.33, 0.95)∗
Smoking, number of cigarettes per day			0.03 (0.002, 0.05)∗
Sleep time, number of hours per day			−0.17 (−0.24, −0.10)∗
Change in household members			0.10 (−0.11, 0.31)
No outdoor spaces at home			−0.09 (−0.39, 0.21)
Worse financial management			0.70 (0.49, 0.90)∗
Social support,^b^ range 3–9			−0.44 (−0.56, −0.31)∗
Loneliness,^c^ range 4–12			1.00 (0.95, 1.06)∗
Effect size, Cohen's f^2^	0.002	0.044	0.461

CI = confidence interval.

Psychological symptoms were measured using the Kessler Distress Scale (K6) (range 0–24).

The model accounted for clustering within adolescence.

∗Significant at *p* < ·05.

a Mental difficulties were measured using the Strength and Difficulties Questionnaire.

b Social support was measured using a 3-item version of the Social Provisions Scale.

c Loneliness was measured using a 4-item UCLA Loneliness Scale.

The results of a sensitivity analysis, in which individuals with missing data were excluded from the fully adjusted model, did not meaningfully differ in association with K6 scores or psychosocial risk factors, except for police arrest (coefficient = −1.76; 95% confidence interval [95% CI] = −3.56, .03).

### Association between caring status and mental well-being

Regarding the WEMWBS, carers showed significantly lower mean scores than noncarers after exposure in the unadjusted model (Table 4). WEMWBS scores at wave 3 were significantly lower than those at wave 1. In the model adjusted for baseline features, caring status no longer showed significant associations with WEMWBS scores after exposure. The fully adjusted model with psychosocial variables after exposure showed significant associations with lower mental well-being at wave 3 than at wave 1. The psychosocial variables include older age, female gender, poor sleep quality, suicide attempt at baseline, increased alcohol consumption, decreased sleep time, change in household members, outdoor spaces at home, poor financial management, less social support, and greater feelings of loneliness at exposure. Caring status at baseline was not significantly associated with mental well-being after exposure. The local effect size was small in the unadjusted model and the model adjusted for baseline features. However, it was large in the fully adjusted model (Table 4).

**Table 4:** Multiple linear regression analyses of mental well-being controlling for baseline and exposure variables

Coefficient (95% CI)	Mental well-being (WEMWBS)^b^		
	Unadjusted	Adjusted	Fully adjusted
Carer at baseline	–0.65 (–1.02, –0.27)∗	–0.30 (–0.67, 0.08)	–0.002 (–0.28, 0.28)
Time, reference = wave 1			
Wave 2	0.16 (–0.01, 0.34)	0.13 (–0.05, 0.30)	0.23 (0.06, 0.40)∗
Wave 3	–0.62 (–0.79, –0.45)∗	–0.69 (–0.86, –0.52)∗	–0.42 (–0.58, –0.26)∗
Outcome at baseline	0.43 (0.40, 0.45)∗	0.33 (0.30, 0.36)∗	0.21 (0.19, 0.24)∗
Demographic at baseline			
Age, year		0.27 (–0.03, 0.57)	0.26 (0.02, 0.50)∗
Female		–0.81 (–1.04, –0.59)∗	–0.94 (–1.13, –0.76)∗
Non-White Caucasian		0.15 (–0.15, 0.44)	0.10 (–0.14, 0.35)
Psychosocial risk factors at baseline			
Heavy drinking		0.32 (–0.05, 0.69)	0.22 (–0.09, 0.54)
Regular smoking		–0.18 (–0.76, 0.40)	–0.06 (–0.51, 0.40)
Cannabis use, reference = never			
Less than 10 times		0.10 (–0.15, 0.34)	–0.01 (–0.22, 0.21)
10 or more times		0.30 (–0.25, 0.85)	0.04 (–0.39, 0.47)
Use of other drugs		–0.44 (–0.94, 0.06)	–0.20 (–0.57, 0.18)
Poor sleep quality		–0.89 (–1.10, –0.67)∗	–0.27 (–0.45, –0.10)∗
Being arrested		0.55 (–0.68, 1.79)	0.93 (–0.15, 2.02)
Problematic video gaming		0.06 (–0.26, 0.38)	0.11 (–0.18, 0.39)
Gambling		–0.13 (–0.44, 0.18)	–0.18 (–0.43, 0.07)
Self-harm		–0.56 (–0.79, –0.33)∗	0.13 (–0.05, 0.32)
Suicide attempt		–0.52 (–0.88, –0.15)∗	–0.37 (–0.67, –0.07)∗
Mental difficulties,^a^ range 0–40		–0.05 (–0.07, –0.02)∗	–0.01 (–0.03, 0.01)
Psychosocial risk factors after exposure			
Alcohol consumption, number of drinks per day, reference = never			
1–2			–0.01 (–0.23, 0.22)
3–4			–0.11 (–0.35, 0.14)
5 or more			–0.30 (–0.59, –0.05)∗
Smoking, number of cigarettes per day			–0.01 (–0.02, 0.01)
Sleep time, number of hours per day			0.10 (0.06, 0.15)∗
Change in household members			–0.20 (–0.34, –0.05)∗
No outdoor spaces in house			0.29 (0.07, 0.52)∗
Worse financial management			–0.47 (–0.62, –0.32)∗
Social support,^b^ range 3–9			0.50 (0.42, 0.57)∗
Loneliness,^c^ range 4–12			–0.68 (–0.72, –0.64)∗
Effect size, Cohen's f^2^	0.002	0.045	0.428

CI = confidence interval; WEMWBS = Warwick-Edinburgh Mental Well-being Scale.

Mental well-being was measured using the Warwick-Edinburgh Mental Well-being Scale (range 7–35).

The model accounted for clustering within adolescence.

∗Significant at *p* <·05.

a Mental difficulties were measured using the Strength and Difficulties Questionnaire.

b Social support was measured using a 3-item version of the Social Provisions Scale.

c Loneliness was measured using a 4-item UCLA Loneliness Scale.

In another sensitivity analysis that excluded individuals with missing data, associations with WEMWBS scores were not significant for age at baseline (coefficient = −.21; 95% CI = −.15, .56), alcohol consumption (coefficient = −.26; 95% CI = −.58, .07), and outdoor spaces at home after exposure (coefficient = .12; 95% CI = −.20, .44). Other results did not differ significantly.

## Discussion

In this cohort study, we investigated the longitudinal mental health outcomes of adolescent carers from the age of 17 years until one year after the first national lockdown in the UK. Carers consistently reported significantly worse psychological symptoms and mental well-being than noncarers during the pandemic. However, these associations were explained by the psychosocial risk factors at baseline and exposure. Worse outcomes were associated with poor sleep quality and suicide attempts at 17 years of age, as well as low social support and strong feelings of loneliness during the pandemic. Adolescent carers were significantly more likely to experience these psychosocial risk factors than noncarers. The local effect size per model showed that mental health outcomes were largely explained by psychosocial risk factors during the pandemic.

The aforementioned existing inequalities among carers at 17 years of age appear to have affected mental health outcomes during the pandemic. Carers may have lived with the negative impacts of their caring role, unaddressed health and behavior problems, and lack of access to required social support services [9,10]. Although school closures saved commuting time and homework, being confined to the home increased difficulty in balancing caring responsibilities [30,31]. Financial hardship could have added to the psychological distress of adolescent carers who were unable to access social support services owing to COVID-19–related restrictions [13,14]. Furthermore, adolescents have specific needs for their social and emotional development. Young carers were first included and defined in the UK legislation in 2014. Therefore, the UK has advanced awareness and policy responses for young carers [[15], [16], [17]]. The voluntary sector has historically provided more support for them compared to the governmental sector. However, such support is decreasing owing to reduced funding [17]. During the pandemic, young carers reported increased worries and concerns regarding the health and well-being of their care receivers—individuals at high risk from COVID-19 [13,30]. Psychosocial support for adolescent carers should be strengthened to address needs that are modifiable, despite COVID-19–related restrictions. For example, online psychoeducational sessions have been developed using video conference instruments, to build psychological resilience among young carers [32].

Similarly, carers reported receiving significantly lower social support and experiencing a greater feeling of loneliness during the pandemic. These factors were significantly associated with worse mental health outcomes in this study, as has been shown in general adolescent populations [7,33,34]. The adverse effects of school closures and loss of time away from home were exacerbated for those who take up caring roles in adolescence. While young carers reported that COVID-19 provided them more time to spend with their care receivers and enhance their relationships [30], many young carers reported feeling less connected to others than they did before the pandemic [14]. Notably, with the rapid vaccine rollout, the UK removed restrictions from spring 2021. As schools reopened, young carers may have realized their differences from students who did not take up caring roles and may have experienced a wider range of emotions. Reopening of schools could also invoke feelings of being behind in educational attainment or skills development that have been exacerbated by the transition to online learning modalities [30]. Educational settings require careful consideration of support regarding the school curriculum, returning to school, and catching up with friends in person.

The overall sample showed increased psychological symptoms and decreased mental well-being from the pre–COVID-19 to current COVID-19 period. Poorer mental health outcomes were observed among females, with increased alcohol consumption, decreased sleep time, and poor financial management. These associations are consistent with previous studies regarding adolescent mental health during the pandemic [4,23]. Our study confirmed findings for these risk factors by including outcome measures at the age of 17 years as a baseline in the pre–COVID-19 period. Notably, based on the fully adjusted model, mental well-being at wave 3 was even worse than that at wave 1, when the first national lockdowns were implemented at the highest level. The decreased mental health in this sample may have been a long-term consequence rather than an immediate response to pandemic restrictions [3]. Being arrested by the police during the pre–COVID-19 period was significantly associated with lower psychological symptoms. These adolescents may have received some form of follow-up contact and support, which eventually moderated the impact of COVID-19 restrictions.

The strength of our study lies in the use of a representative cohort study in the UK. The longitudinal design, including the MCS sweep 7 survey and three waves of the COVID-19 survey, elucidated the long-term impacts of the pandemic on adolescent mental health outcomes. However, our study has some limitations. Caring status at 17 years of age could have varied during the pandemic because of change in family members' care location, such as hospital admission and nursing home placement. The definition of caring status could have excluded adolescents who cared for young siblings because of their parent's illness or disabilities. Our analyses did not consider parents' economic adversities, which may also affect adolescent caring status and mental health outcomes. Additionally, owing to missing data, our analyses did not include information on socioeconomic status, school attendance, and level of worry about family members' health. These variables could have confounding associations between caring status and mental health outcomes. Although the COVID-19 survey included assessment of caring in households, the questions varied across the wave 1 survey and later waves. Thus, we could not determine whether caring continued or not or who was new to caring responsibilities during exposure to the COVID-19 pandemic. Future studies should investigate mental health consequences among new young carers during COVID-19–related restrictions.

Despite these limitations, our study indicated that a caring role was significantly associated with worsening mental health outcomes among adolescents during the COVID-19 pandemic. Our results highlight the need for psychosocial support for young carers. As the UK has been removing COVID-19–related restrictions since spring 2021, careful consideration is warranted to support adolescent carers to return to school and catch up with studies and other students.

## Data Statement

University of London, Institute of Education, Centre for Longitudinal Studies owns the copyright for the Millennium Cohort Study (MCS) data used in this study (2021). Millennium Cohort Study: Seventh Survey, 2018. [Data Collection]. UK Data Service. SN: 8682, https://doi.org/10.5255/UKDA-SN-8682-1.

## Supplementary Data

Appendix A1
