# Supplementary material for: Adolescent Carers' Psychological Symptoms and Mental Well-being During the COVID-19 Pandemic: Longitudinal Study Using Data From the UK Millennium Cohort Study
Source: J Adolesc Health. 2022 Jun;70(6):877–84. doi: 10.1016/j.jadohealth.2022.01.228 (PMC8813552; doi:10.1016/j.jadohealth.2022.01.228)
Supplement: Appendix A1 [file mmc1.docx]

**Appendix**

**Table A1. Data sources and variables from the Millennium Cohort Study and COVID-19 survey**

| Survey | Sample | Variables |
| --- | --- | --- |
| Baseline  MCS sweep 7, age 17  January 2018–March 2019 | 3,927 | **Primary exploratory variable**   - Caring status   **Outcome measures**   - Psychological symptoms (K6) - Mental well-being (WEMWBS)   **Demographic**   - Age, gender, and ethnicity   **Psychosocial risk factors**   - Heavy drinking (10 or more episodes involving at least 5 drinks at a time in the last year) - Regular smoking (more than 5 cigarettes a week in the last yar) - Cannabis use (no use; less than 10 times in the last year; 10 times or more) - Use of other drugs (at least once in the last year) - Subjective sleep quality over the last year - Being arrested by a police officer and taken to a police station in the last year - Problematic video gaming (5 or more hours on a normal weekday) - Gambling (having spent money on either fruit machines, betting at a betting shop, or online gambling in last month) - Self-harm (Self-bruising, pulling out their hair or cutting, or taking an overdose of tablets during the last year) - Suicide attempt (having hurt themselves on purpose to end their life) - Mental health difficulties evaluated by parent using the Strengths and Difficulties Questionnaire (SDQ) |
| Exposure, COVID-19   - Wave 1, May 2020 | 1,946 | **Outcome measures**   - Psychological symptoms (K6) - Mental well-being (WEMWBS)   **Psychosocial risk factors**   - Alcohol consumption (number of drinks a day) - Smoking (number of cigarettes a day) - Sleep time (number of hours per day) - Change in the household member composition from the pre-lockdown period - Having outdoor spaces at home - Financial management (worse) - Social support (Social Provisions Scale) - Loneliness (UCLA Loneliness Scale) |
| - Wave 2, September–October 2020 | 2,429 |  |
| - Wave 3, February–March 2021 | 3,263 |  |

K6: K6 Kessler Distress Scale; WEMWBS: Warwick-Edinburgh Mental Wellbeing Scale
